# miR-30a suppresses lung cancer progression by targeting SIRT1

**DOI:** 10.18632/oncotarget.23529

**Published:** 2017-12-21

**Authors:** Yaowu Guan, Zhongming Rao, Cheng Chen

**Affiliations:** ^1^ Department of Thoracic Surgery, Zhumadian Central Hospital, Zhumadian, Henan 463000, China; ^2^ Department of Radiotherapy, Nanjing Medical University Affiliated Cancer Hospital, Cancer Institute of Jiangsu Province, Nanjing, Jiangsu 210009, China

**Keywords:** miR-30a, SIRT1, proliferation, apoptosis, invasion

## Abstract

The class III histone deacetylase silent information regulator 1 (SIRT1) is frequently overexpressed in a variety of tumors, including lung cancer; however, its regulatory mechanisms are largely unknown. In this study, we found that an inconsistent trend between SIRT1 protein and mRNA levels in human lung cancer tissues, suggesting that a post-transcriptional mechanism may involved in SIRT1 regulation. Because microRNAs are important post-transcriptional regulators of gene expression, candidate miRNAs that could potentially bind SIRT1 were gained through bioinformatics analyses. We further experimentally validated SIRT1 as the direct target of miR-30a by evaluating SIRT1 expression in lung cancer cells after the overexpression or knockdown of miR-30a and by luciferase assay. Moreover, we showed that miR-30a inhibited proliferation, invasion and promoted apoptosis of lung cancer cells by inhibiting SIRT1 *in vitro* and *in vivo*. Taken together, this study identified a new regulatory axis in which miR-30a and SIRT1 regulate the proliferation, invasion and apoptosis of lung cancer cells and lung tumorigenesis.

## INTRODUCTION

Lung cancer is the most frequently diagnosed and leading cause of death cancer worldwide [1], and non-small-cell lung cancer (NSCLC) accounts for 75–80% of all cases [2]. Most lung cancer patients are diagnosed with metastatic and advanced disease, and only a small proportion is eligible for surgical resection and radical treatment [3]. Thus, new treatment strategies are needed, and further understanding the molecular mechanisms underlying lung carcinogenesis is of major significance and might provide novel strategies for lung cancer treatment.

Sirtuins are belong to the class III histone deacetylase (HDAC) family and have been linked to longevity in lower organisms and to mammalian metabolism [4, 5]. Sirtuin 1 (SIRT1) functions by deacetylating histone (e.g. H4-Lys16 and H3-Lys9) and non-histone proteins(e.g. Ku70 and p300) in an NAD^+^-dependent manner, thus modifying gene expression and modulating protein activity [6, 7]. SIRT1 plays important roles in numerous processes, including cell cycle, metabolism, DNA repair, aging and cell survival under stress conditions [4, 5].

Importantly, dysregulation of SIRT1 has been demonstrated in various cancers including prostate, breast, ovarian and lung cancers, implicating a pathogenetic role for SIRT1 in malignancies [8–11]. SIRT1 can function as an oncogene by regulating the acetylation of some tumor suppressors, including p53 [12] and FoxOs [13]. However, other studies also suggest that SIRT1 may also have tumor-suppressive function in some mouse model [14, 15]. The precise role of SIRT1 in cancer may depend on the specific cell or tumor type. Though up-regulation of SIRT1 has been observed in lung cancer [11], the roles of SIRT1 in the initiation and progression of lung cancer remain poorly understood.

SIRT1 expression can be regulated at the transcriptional level. For example, tumor suppressors p53 and HIC1 (hypermethylated in cancer 1) can suppress SIRT1 transcription through binding to SIRT1 promoter region [16, 17]. However, this is not the unique mechanism for dysregulation of SIRT1 in tumors. For example, HuR, a RNA binding protein, stabilizes SIRT1 mRNA through 3′-untranslated region (3′-UTR) interactions leading to increased SIRT1 levels (19). This suggests that the post-transcriptional regulation of SIRT1 may also be significant in governing SIRT1 expression in lung cancer.

MiRNAs are small non-coding RNAs of 20∼22 nucleotides which can lead to mRNA degradation and/or translational repression repress gene expression through interactions with the 3′-untranslated regions (3′-UTRs) of target gene transcripts [18–20]. Through this mechanism, miRNAs can regulate a great variety of biological processes, including cell proliferation, differentiation, migration, apoptosis, development and metabolism [21–23]. MiR-30a has been reported significantly down-regulated in lung cancer [24], indicating that miR-30a may play an important role in tumorigenesis and development of lung cancer. However, the function of miR-30a, especially in lung cancer, remains unclear.

In this study, we showed that SIRT1 as a direct target gene of miR-30a, resulting in the downregulation of SIRT1 protein expression. The potential role of miR-30a as an anti-oncogene of lung cancer through SITR1 targeting in proliferation, invasion and apoptosis had been experimentally validated *in vitro* and *in vivo*.

## RESULTS

### The upregulation of SIRT1 protein but not mRNA in lung cancer tissues

**Figure 1:**
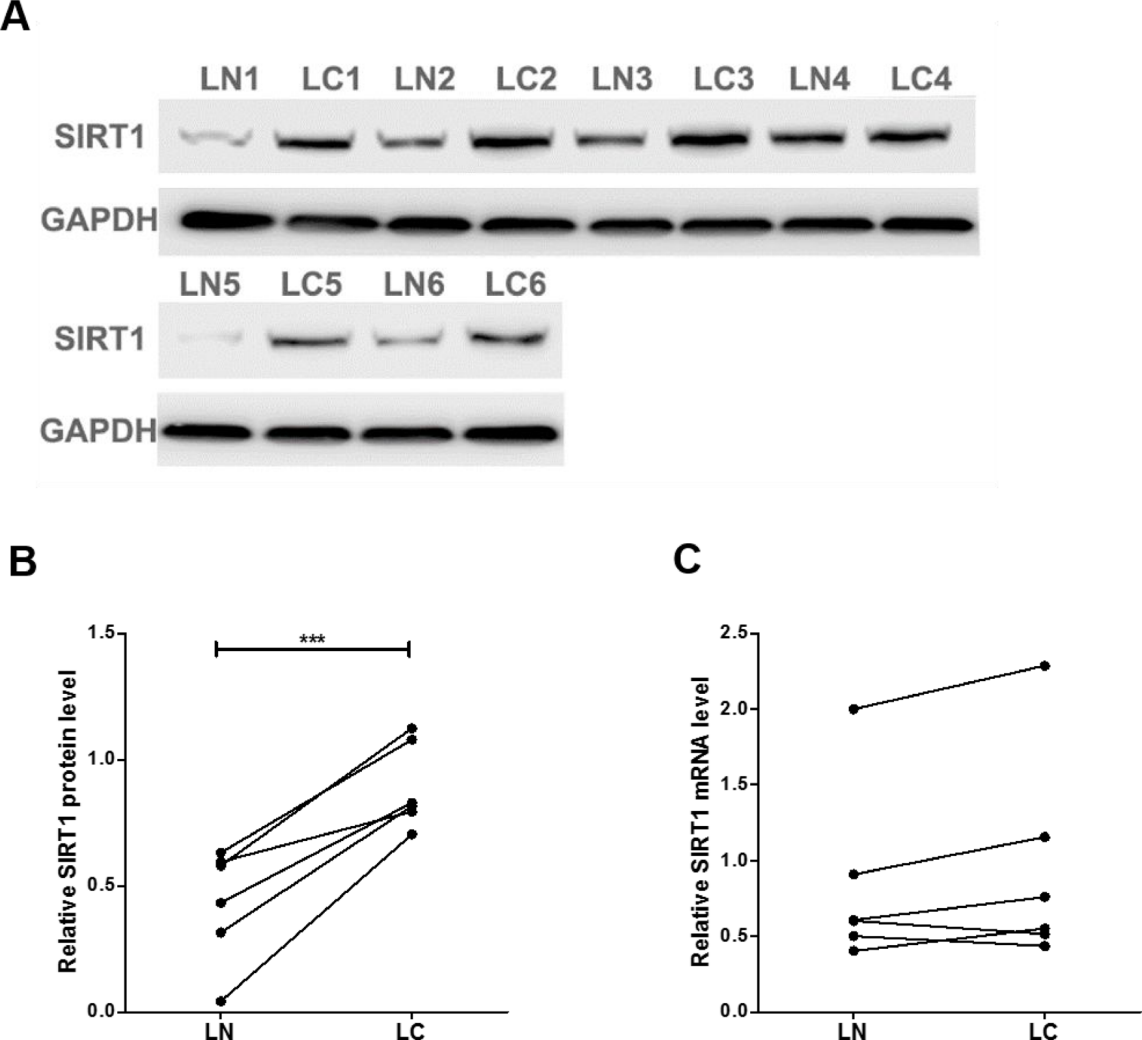
SIRT1 protein and mRNA expression levels in lung cancer tissues (**A** and **B**) Western blot analysis of the expression of the SIRT1 protein in 6 pairs of lung cancer (LC) and normal adjacent (LN) tissue samples. A: representative image; B: quantitative analysis. (**C**) Quantitative RT-PCR analysis of the relative expression levels of SIRT1 mRNA in 6 pairs of LC and LN samples. ^***^P < 0.001.

We first determined the SIRT1 protein levels in 6 pairs of clinical lung cancer tissues. We found that SIRT1 protein levels were consistently upregulated in lung cancer tissues (Figure 1A and 1B). Subsequently, we performed quantitative RT-PCR to check the levels of SIRT1 mRNA in the same 6 pairs of cancerous and noncancerous tissues. However, SIRT1 mRNA levels did not significantly differ between cancerous and noncancerous tissues (Figure 1C). The disparity between SIRT1 protein and mRNA levels in lung cancer tissues suggested a post-transcriptional mechanism involved in the regulation of SIRT1.

### Prediction of SIRT1 as a target of miR-30a

**Figure 2:**
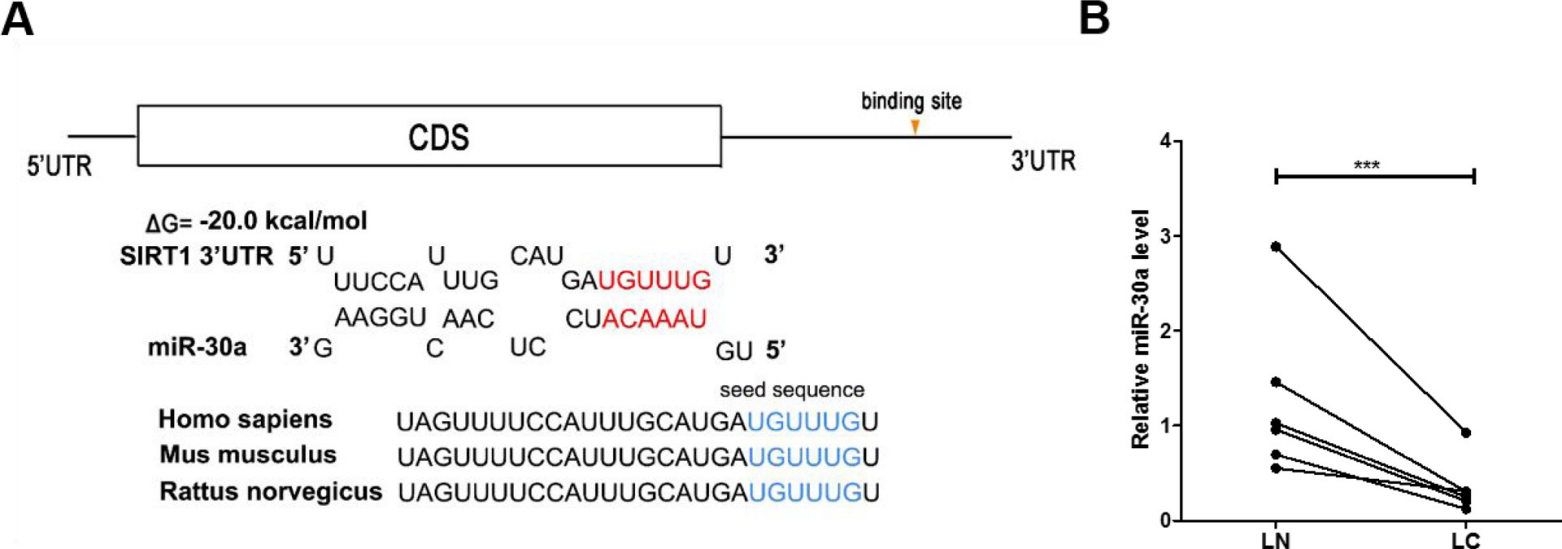
Prediction of the miR-30a binding site within the SIRT1 3′-UTR (**A**) Schematic depicting the hypothetical duplexes formed through interactions between the binding sites in the SIRT1 3′-UTR (top) and miR-30a (bottom). The predicted free energy of each hybrid is indicated. The seed recognition sites are denoted, and all nucleotides in these regions are highly conserved across species. (**B**) Quantitative RT-PCR analysis of the miR-30a expression levels in six pairs of LC and LN samples. ^***^P < 0.001.

One common post-transcriptional regulation is the suppression of mRNA transcripts by miRNAs. To identify which miRNAs can potentially target SIRT1 in lung cancer cells, a list of predicted miRNAs which may target SIRT1 was compiled using three computational algorithms, TargetScan [25], miRanda [26] and PicTar [27]. Among the candidates, miR-30a is a tumor suppressor that is frequently downregulated in lung cancer [28, 29]. The predicted interactions between miR-30a and the targeting sites within the 3′-UTR of SIRT1 are illustrated in Figure 2A.

### Detection of an inverse correlation between miR-30a and SIRT1 levels in lung cancer tissues

Based on the opposite expression patterns between miRNAs and their target genes, we next investigated whether miR-30a was inversely correlated with SIRT1 in clinical patient tissues. After measuring the miR-30a levels in 6 pairs of lung cancer tissues and noncancerous tissues, we observed that miR-30a was indeed downregulated in lung cancer tissues (Figure 2D). We suggested that SIRT1 was a target of miR-30a, based on both computational predictions and the inverse correlation between miR-30a and SIRT1 levels in human lung cancer tissues.

### Validation of SIRT1 as a direct target of miR-30a

**Figure 3:**
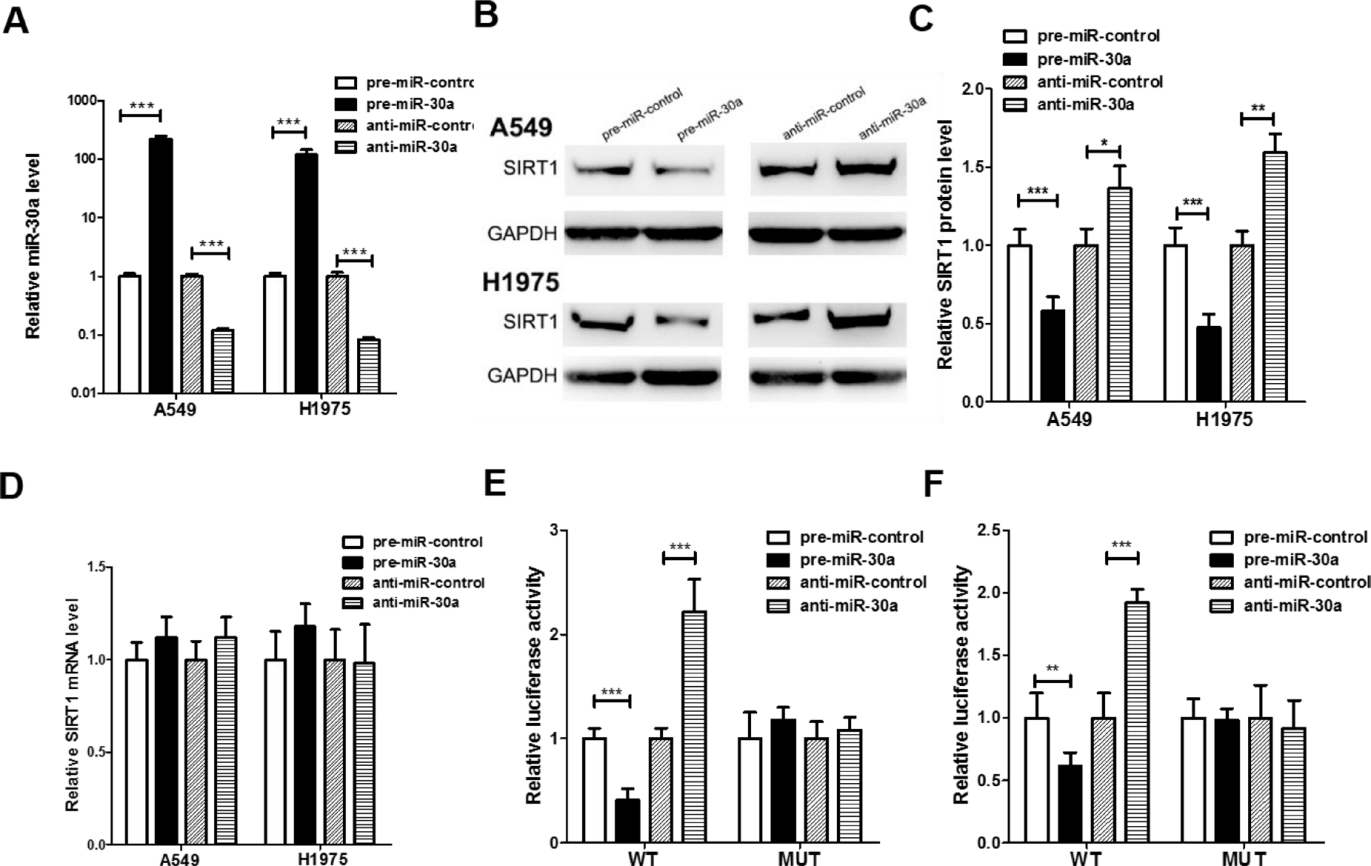
Direct post-transcriptional regulation of SIRT1 expression through miR-30a (**A**) Quantitative RT-PCR analysis of the expression levels of miR-30a in A549 and H1975 cells transfected with equal doses of the miR-30a mimic (pre-miR-30a), miR-30a inhibitor (anti-miR-30a) or scrambled negative control RNA (pre-miR-control or anti-miR-control). (**B** and **C**) Western blotting analysis to detect SIRT1 protein levels in A549, and H1975 cells transfected with equal doses of the miR-30a mimic, miR-30a inhibitor or scrambled negative control RNA. (B) representative image; (C) quantitative analysis. (**D**) Quantitative RT-PCR analysis of SIRT1 mRNA levels in A549 and H1975 cells transfected with equal doses of the miR-30a mimic, miR-30a inhibitor or scrambled negative control RNA. (**E** and **F**) Direct recognition of the SIRT1 3′-UTR by miR-30a. Firefly luciferase reporters containing either wild-type (WT) or mutant (MUT) miR-30a binding sites in the SIRT1 3′-UTR were co-transfected into 293T (E) and A549 (F) cells with equal doses of the miR-30a mimic, miR-30a inhibitor or scrambled negative control RNA. After twenty-four hours post-transfection, the cells were assayed using a luciferase assay kit. Firefly luciferase values were normalized to β-galactosidase activity, and the results were calculated as the ratio of firefly luciferase activity in the miR-30a-transfected cells normalized to the negative control RNA-transfected cells. The results are presented as the mean ± S.E. of three independent experiments. (^*^p < 0.05; ^**^p < 0.01; ^***^p < 0.005).

Two human lung cancer cell lines (A549 and H1975) was used to further confirm the direct correlation between miR-30a and SIRT1 after overexpression or knockdown of miR-30a. As expected, the cellular levels of miR-30a were significantly increased in A549 and H1975 cells transfected with miR-30a mimics and decreased dramatically when cells were transfected with miR-30a inhibitor (Figure 3A). Consequently, the expression of SIRT1 protein was significantly inhibited by the overexpression of miR-30a in A549 and H1975 cells, while the miR-30a inhibitor significantly increased the SIRT1 protein levels in lung cancer cells (Figure 3B and 3C). To determine at what level miR-30a influenced SIRT1 expression, we repeated the above experiments and examined the expression of SIRT1 mRNA after transfection. Overexpression or knockdown of miR-30a did not affect SIRT1 mRNA levels (Figure 3D).

A luciferase reporter assay was performed to confirm whether the negative regulatory effects of miR-30a on SIRT1 expression were mediated by the binding of miR-30a to the predicted sites in the 3′-UTR of SIRT1 mRNA and inhibit SIRT1 expression. The full-length 3′-UTR of SIRT1, containing the presumed miR-30a binding sites, was placed downstream of the firefly luciferase gene in a reporter plasmid. Then the recombination plasmid was transfected into 293T and A549 cells along with pre-miR-30a. As expected, the luciferase activity was dramatically reduced in cells co-transfected with luciferase reporter plasmid and miR-30a mimics (Figure 3E and 3F). Then we introduced point mutations into the binding site of the SIRT1 3′-UTR to eliminate the predicted miR-30a binding site. This mutated luciferase reporter was unaffected through either the overexpression or knockdown of miR-30a (Figure 3E and 3F). This finding suggested that the binding sites strongly contribute to the interaction between miR-30a and SIRT1 mRNA. In conclusion, our results demonstrate that miR-30a directly recognizes and binds to the 3′-UTR of the SIRT1 transcript and inhibits SIRT1 translation.

### The role of miR-30a in regulating SIRT1 in lung cancer cells

**Figure 4:**
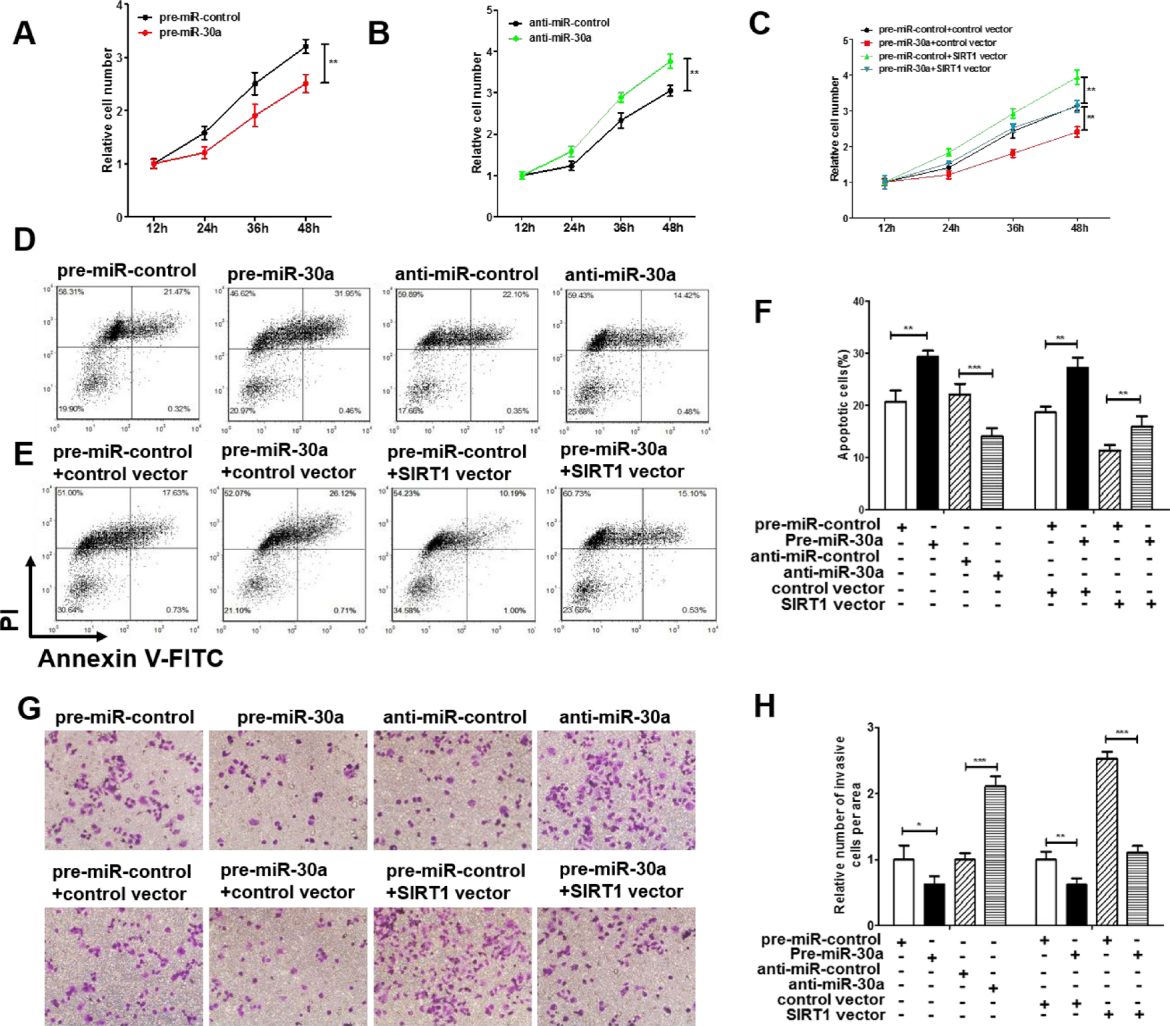
Effect of miR-30a and SIRT1 on the proliferation and apoptosis of lung cancer cells (**A**) A cell proliferation assay was performed 12, 24, 36 and 48 hours after the transfection of A549 cells with equal doses of the miR-30a mimic or scrambled negative control RNA. (**B**) The cell proliferation assay was performed 12, 24, 36 and 48 hours after the transfection of A549 cells with equal doses of the miR-3a inhibitor or scrambled negative control RNA. (**C**) The cell proliferation assay was performed 12, 24, 36 and 48 hours after the transfection of A549 cells with equal doses of the pre-miR-control plus control plasmid, pre-miR-control plus SIRT1 overexpression plasmid, miR-30a mimic plus control plasmid, or miR-30a mimic plus SIRT1 overexpression plasmid. (**D–F**) The apoptosis assay was performed 24 hours after the transfection of A549 cells with equal doses of the miR-30a mimic, miR-30a inhibitor or scrambled negative control RNA or with equal doses of the pre-miR-control plus control plasmid, pre-miR-control plus SIRT1 overexpression plasmid, miR-30a mimic plus control plasmid, or miR-30a mimic plus SIRT1 overexpression plasmid. (D and E) representative image; (F) quantitative analysis. (**G** and **H**) Transwell analysis was performed after the transfection of A549 cells with equal doses of the miR-30a mimic, miR-30a inhibitor or scrambled negative control RNA or with equal doses of the pre-miR-control plus control plasmid, pre-miR-control plus SIRT1 overexpression plasmid, miR-30a mimic plus control plasmid, or miR-30a mimic plus SIRT1 overexpression plasmid. G. representative image; H. quantitative analysis. The results are presented as the mean ± S.E. of three independent experience (^*^p < 0.05; ^**^p < 0.01; ^***^p < 0.005).

We next analyzed the biological function of miR-30a-driven repression of SIRT1 expression in lung cancer cells. Because SIRT1 is known to induce cell proliferation [30] and invasion [31] and suppress apoptosis [32], miR-30a may suppress SIRT1 expression to affect cell proliferation, invasion and apoptosis. As expected, overexpression of miR-30a in A549 cells showed decreased cell viability and invasion and increase cell apoptosis; in contrast, inhibiting miR-30a had an opposite effect on lung cell proliferation, invasion and apoptosis (Figure 4A, 4B, 4D, 4F, 4G and 4H). Efficient overexpression of SIRT1 in A549 cells was achieved (Supplementary Figure 1A–1F). As expected, overexpression of SIRT1 in A549 cells indeed caused increased cell proliferation, invasion and decreased cell apoptosis (Supplementary Figure 2A–2E). More importantly, proliferation, invasion and apoptosis assays revealed that ectopic expression of miR-30a-resistant SIRT1 dramatically attenuated the inhibitory effect of the miR-30a on cell proliferation, invasion and apoptosis (Figure 4C, 4E, 4F, 4G and 4H). Taken together, our results suggest that SIRT1 is crucial to the proliferation, invasion and apoptosis of lung cancer cells and that miR-30a might suppress proliferation and invasion and promote apoptosis in lung cancer cells by silencing SIRT1.

### The function of miR-30a-SIRT1 axis on lung cancer growth *in vivo*

**Figure 5:**
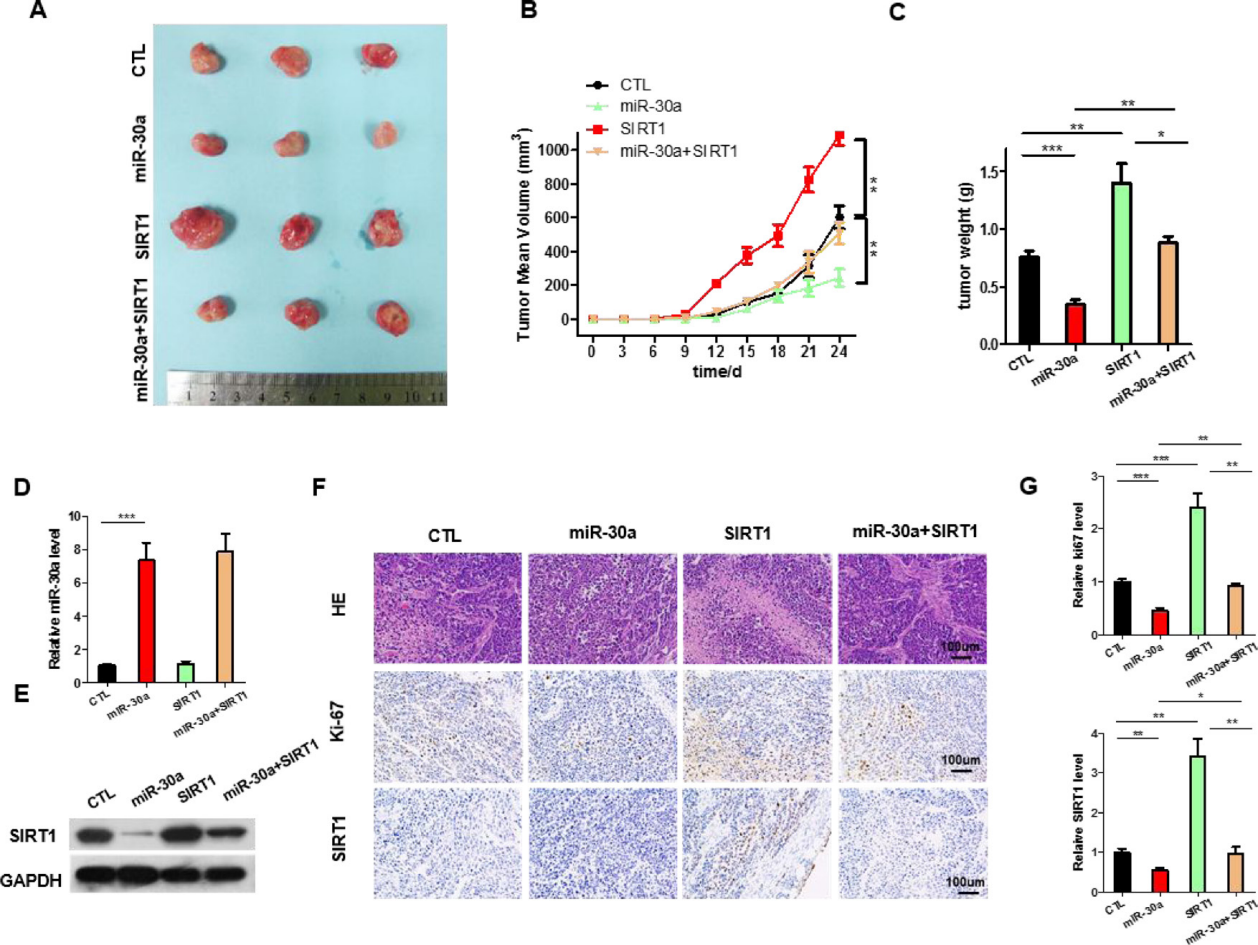
The function of miR-30a-SIRT1 axis on lung cancer growth *in vivo.* Mice were divided into four groups according to the implanted A549 cells: control cells (CTL), miR-30a overexpressing cells (miR-30a), SIRT1-overexpressing cells (SIRT1), and SIRT1 plus Lenti-miR-30a cells (miR-30a+ SIRT1). (**A**) Representative images of tumors. (**B**) The time course of tumor volume. (**C**) The quantitative analysis of tumor weight. (**D**) Quantitative RT-PCR analysis of the expression levels of miR-30a in tumors from four groups of mice. (**E**) Western blotting analyses of SIRT1 proteins in tumors from four groups of mice. (**F**) HE staining, Ki67 and SIRT1 immunohistochemical staining of tumor tissues in four groups of mice. (**G**) Quantitative analyses of Ki-67-positive and SIRT1-positive signals in the tumor. All data are shown as the means ± S.E. obtained from three separate experiments. (^*^p < 0.05; ^**^p < 0.01; ^***^p < 0.005).

We next evaluated the effects of miR-30a and SIRT1 on the growth of lung cancer xenograft in mice. A549 cells were infected with the lentiviral plasmid to express miR-30a. Efficient overexpression of miR-30a and inhibition of SIRT1 was shown in Supplementary Figure 3A–3B. The A549 cells (1 × 10^6^ cells per 0.1 mL) were infected with/without the miR-30a lentiviral expression plasmid (miR-30a), SIRT1 plasmid(SIRT1), or miR-30a lentiviral expression plasmid plus SIRT1 overexpression plasmid(miR-30a+SIRT1); then, the cells were implanted subcutaneously into 6-week-old nude mice and tumor growth was measured after 25 days. A significant decrease in the sizes and growth of tumors was observed in the miR-30a-overexpressing group compared with the control group, whereas the sizes and weights in the tumors from the group implanted with the SIRT1-overexpression plasmid were dramatically increased (Figure 5A–5C). Additionally, SIRT1 overexpression attenuated the suppressive effect of miR-30a on tumor growth (Figure 5A–5C), suggesting that miR-30a might inhibit tumor growth by silencing SIRT1. After 25 days, the miR-30a-overexpression group show a significant increase in the expression of miR-30a compared with tumors from control group (Figure 5D). Tumors from miR-30a-overexpressing group displayed reduced SIRT1 protein levels compared with tumors from control group, whereas the tumors of SIRT1-overexpressing group showed elevated SIRT1 protein levels (Figure 5E). Tumors with both miR-30a plus SIRT1 overexpression exhibited significantly higher levels of SIRT1 compared to tumors with miR-30a overexpression (Figure 5E), suggesting that SIRT1 overexpression is sufficient rescue the SIRT1 suppression caused by miR-30a. Furthermore, Hematoxylin and eosin (H&E) staining of xenograft tissues showed more cell mitosis in the SIRT1-overexpressing group than the control group (Figure 5F). Xenografts from miR-30a plus SIRT1 overexpression group exhibited less cell mitosis compared with xenografts with SIRT1 overexpression (Figure 5F and 5G), suggesting that miR-30a overexpression could attenuate the pro-proliferative effect of SIRT1. Immunohistochemical staining also revealed the lower levels of SIRT1 in tumors from mice implanted with miR-30a-overexpressing cells, whereas the tumors from the SIRT1-overexpressing mice showed increased SIRT1 protein levels (Figure 5F and 5G). Finally, the proliferative activity of tumor cells were assessed by immunocytochemistry with the mouse monoclonal antibody Ki-67. The cell proliferation rate measured by the percentage of Ki-67-positive tumor cells were increased in the group implanted with the SIRT1 plasmids and decreased in the group implanted with the miR-30a lentivirus (Figure 5F and 5G). Likewise, miR-30a overexpression attenuated the pro-proliferative effect caused by SIRT1 overexpression (Figure 5F). These results validated the role of miR-30a in regulating lung cancer tumorigenesis.

## DISCUSSION

Lung cancer is one of the most common cancers worldwide, and NSCLC constitutes approximately 80% of all primary lung cancers [33]. The efficacy of new treatments remains limited by a combination of drug resistance and our insufficient understanding of tumor cell signaling pathways. Many genes, including cancer inhibitors (tumor suppressors) and cancer inducers (oncogenes), influence lung carcinogenesis. SIRT1, a conserved nicotinamide adenine dinucteotide (NAD(+))-dependent deacetylase, has been implicated in the occurrence and development of lung cancer. Noh *et al*. confirmed that SIRT1 was significantly up-regulated in lung cancer [11]. Sun *et al*. showed that downregulation of SIRT1 by antisense oligonucleotides induces apoptosis and enhances radiation sensitization in lung cancer cells [34]. However, the precise role and regulatory mechanism of SIRT1 in the progression of lung cancer remain poorly understood.

In the present study, we observed that silencing SIRT1 expression through siRNA inhibits proliferation, invasion and promotes apoptosis in lung cancer cells, whereas overexpressing SIRT1 induced the opposite effects, suggesting a role for this protein as an essential oncogene during lung cancer progression. Interestingly, we identified an inconsistent trend between SIRT1 protein and mRNA levels in human lung cancer tissues. These results suggest a post-transcriptional regulation mechanism in regulating SIRT1 expression. One important mode of post-transcriptional regulation is the suppression of mRNA transcripts through miRNAs. Therefore, we searched for potential miRNAs that target SIRT1 and identified miR-30a as a candidate. Mechanistic studies revealed that miR-30a could directly target the SIRT1 3′-UTR and inhibit its expression in lung cancer cells. Moreover, we showed that miR-30a inhibits SIRT1 expression and consequently suppresses proliferation and promotes apoptosis in lung cancer cells. These results indicate a novel regulatory axis in which miR-30a targeting SIRT1 regulates the proliferation and apoptosis of lung cancer cells.

**Figure 6:**
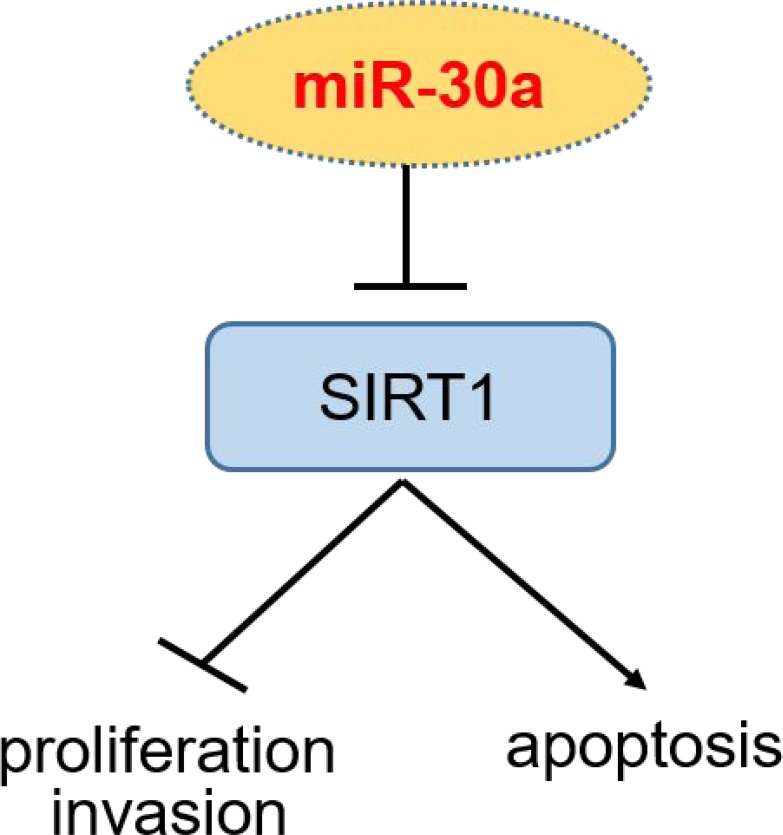
Schematic illustration of the miR-30a-SIRT1 axis

Over the past decade, an important role for miRNAs in the genesis and progression of lung cancers has emerged [35, 36]. In the present study, we observed that the level of miR-30a was lower in lung cancer tissues compared to matched adjacent noncancerous tissue. These results suggest that miR-30a might be involved in the tumorigenesis of lung cancer as a tumor suppressor. Indeed, miR-30a was downregulated in several types of cancers, including lung cancer [29, 37], breast cancer [38], prostate cancer [39] and gastric cancer [40]. Furthermore, miR-30a plays an anti-oncogenic role in cancer through the regulation of proliferation, migration, epithelial-mesenchymal transition and cell adhesion [38, 41–43]. As was shown in Figure 6, we observed that overexpressing miR-30a inhibits proliferation and promote apoptosis in lung cancer cells, and silencing SIRT1 expression mimics miR-30a function *in vitro* and *in vivo*. Interestingly, we found that the restoration of SIRT1 expression successfully attenuates the anti-proliferative and pro-apoptotic effects of miR-30a on lung cancer cells, although miR-30a has many other targets. These results suggest that the targeting of SIRT1 is a main mechanism for miR-30 existing as a tumor suppressor in lung cancer.

Taken as a whole, this study describes a new regulatory axis that miR-30a possesses tumor suppressor activity by negatively regulating SIRT1 expression in lung tumorigenesis. This study may open new view for future lung cancer therapy.

## MATERIALS AND METHODS

### Cells and human tissues

The human lung cancer cell lines, A549 and H1975, were obtained from Shanghai Institute of Cell Biology, Chinese Academy of Sciences(Shanghai, China) and cultured in DMEM (Gibco, Carlsbad, CA, USA) supplemented with 10% fetal bovine serum within a humidified atmosphere containing 5% CO2 at 37°C. The lung tumors and paired normal adjacent tissues were derived from patients undergoing a surgical procedure at the Zhumadian Central Hospital (Zhumadian, China). All protocols concerning the use of patient samples in this study were approved by the Medical Ethics Committee of the Affiliated Zhumadian Central Hospital (Zhumadian, China). A signed consent form was obtained from each donor. The clinical features of the patients are listed in Supplementary Table 1.

### RNA isolation and quantitative RT-PCR

Total RNA extraction, reverse transcription and TaqMan real-time polymerase chain reaction (PCR) for miRNAs were performed according to the manufacturer's instructions as described previously [44]. To quantify SIRT1 mRNA, 1 μg of total RNA was reverse-transcribed to cDNA using oligo dT and Thermoscript (TaKaRa), performed using the following conditions: 42°C for 60 min and 85°C for 5 min. SYBER Green Dye (Invitrogen), and specific primers for SIRT1 and GAPDH were used. The sequences of the primers were as follows: SIRT1 (sense): 5′-CTGTTTCCTGTGGGATACCTGACT-3′; SIRT1 (antisense): 5′-ATCGAACATGGCTTGAGGATCT-3′; GAPDH (sense): 5′-GATATTGTTGCCATCAATGAC-3′; GAPDH (antisense): 5′-TTGATTTTGGAGGGATCTC G-3′. The relative amount of SIRT1 mRNA was normalized to GAPDH.

### miRNA overexpression or knockdown

Synthetic RNA molecules, including pre-miR-30a, anti-miR-30a and scrambled negative control RNAs (pre-miR-control and anti-miR-control) were purchased from GenePharma (Shanghai, China). The cells were seeded onto 6-well plates and transfected using Lipofectamine 2000 (Invitrogen) on the following day when the cells were approximately 70% confluent. In each well, equal 100 pmol of pre-miR-30a, anti-miR-30a or scrambled negative control RNA were used. The cells were harvested at 24 h after transfection for quantitative RT-PCR analysis and Western blotting.

### Plasmid construction and siRNA interference assay

A mammalian expression plasmid encoding the human SIRT1 open reading frame (pReceiver-M02- SIRT1) was purchased from GeneCopoeia (Germantown, MD, USA). An empty plasmid served as a negative control. The siRNAs (siRNA-1: 5′- ACAGUUUCAUAGAGCCAUGAAGUAU-3′, siRNA-2: ACUUUGCUGUAACCCUGUA) targeting human SIRT1 were purchased by GenePharma. A scrambled siRNA (GenePharma) was included as a negative control. Total RNA or protein was isolated 24 h or 48 h after transfection. The SIRT1 protein expression levels were assessed by Western blotting.

### Luciferase reporter assay

A luciferase reporter assay was performed to check the direct binding of miR-30a to the target gene SIRT1 as previously described [45]. The normal and mutant 3′-UTR sequence of SIRT1 was directly synthesized by GenePharma and then inserted into PGL3 plasmid (Ambion). 293T cells were cultured in 24-well plates, and each well was transfected with β-galactosidase (β-gal) expression plasmid (Ambion) and 0.2 μg of firefly luciferase reporter plasmid, and equal amounts of pre-miR-30a and the scrambled negative control RNA using Lipofectamine 2000. The β-gal plasmid was used as a transfection control. After twenty-four hours, the cells were assayed using a luciferase assay kit (Promega, Madison, WI, USA).

### Western blotting

The protein levels were analyzed by Western blot using the appropriate antibodies: anti-SIRT1 antibody (Abcam ab32441, Cambridge, MA, USA) and anti-GAPDH (sc-365062; Santa Cruz Biotechnology, Santa Cruz, CA, USA), and were normalized by probing the same blots with an anti-GAPDH antibody. Protein bands were analyzed using the Image J software.

### Cell proliferation assay

A549 cells were seeded in 96-well plates at a density of 1 × 10^4^ cells per well and transfected 12 hours later. After transfection, 10 μl WST-8 solution from the CCK-8 kit (Beyotime, China) was added into each well. After incubation for 2 hours, the plates were read at 450 nm to measure the absorbance of each well (time 0 hour) and again at 12 h and 60 h. The relative cell number was calculated as the ratio of absorbance of 12 hours to 60 hours.

### Cell invasion and apoptosis assays

The invasion ability and apoptosis of A549 cells was tested in a Transwell Boyden Chamber (6.5 mm, Costar, Corning, NY, USA) and Annexin V-FITC/PI staining kit (BD Biosciences, San Diego, CA, USA). Besides, the total apoptotic cells were counted as the sum of early apoptotic (PI- AV+) and late apoptotic (PI+ AV+) cells.

### Establishment of tumor xenografts in mice

Six-week-old male SCID (severe combined immune deficiency) mice (nu/nu) were purchased from the Model Animal Research Center of Nanjing University (Nanjing, China). Control A549 cells, miR-30a-overexpressing A549 cells, SIRT1- overexpressing A549 cells or miR-30a and SIRT1 co-overexpressing A549 cells were injected subcutaneously into SCID mice (1 × 10^6^ cells per mouse, 3 mice per group). The mice were sacrificed after 25 days. The mouse lung tumors were removed, and the weight of the tumors was measured. Parts of the tissues were used for protein and total RNA extraction, and the remainder were fixed in 4% paraformaldehyde for 24 h and then processed for Hematoxylin and eosin (H&E) staining and immunohistochemical staining for SIRT1 and Ki-67. All animal care and handling procedures were performed in accordance with the National Institutes of Health's Guide for the Care and Use of Laboratory Animals and were approved by the Institutional Review Board of Zhumadian Central Hospital (Zhumadian, China).

### Statistical analysis

All Western blot images are representative of at least three independent experiments. Quantitative RT-PCR, luciferase reporter assays, and cell proliferation and apoptosis assays were performed in triplicate, and each experiment was repeated several times. The data are shown as the means ± SE of at least three independent experiments. The differences were considered statistically significant at *p* < 0.05 using Student's *t*-test.

## SUPPLEMENTARY MATERIALS FIGURES AND TABLES



## Abbreviations

SIRT1: silent information regulator 1
miRNA: microRNA
