# Supplementary material for: miR-30a suppresses lung cancer progression by targeting SIRT1
Source: Oncotarget. 2017 Dec 21;9(4):4924–34. doi: 10.18632/oncotarget.23529 (PMC5797023; doi:10.18632/oncotarget.23529)
Supplement: Supplementary file 1 [file oncotarget-09-4924-s001.pdf]

miR-30a suppresses lung cancer progression by targeting SIRT1

SUPPLEMENTARY MATERIALS

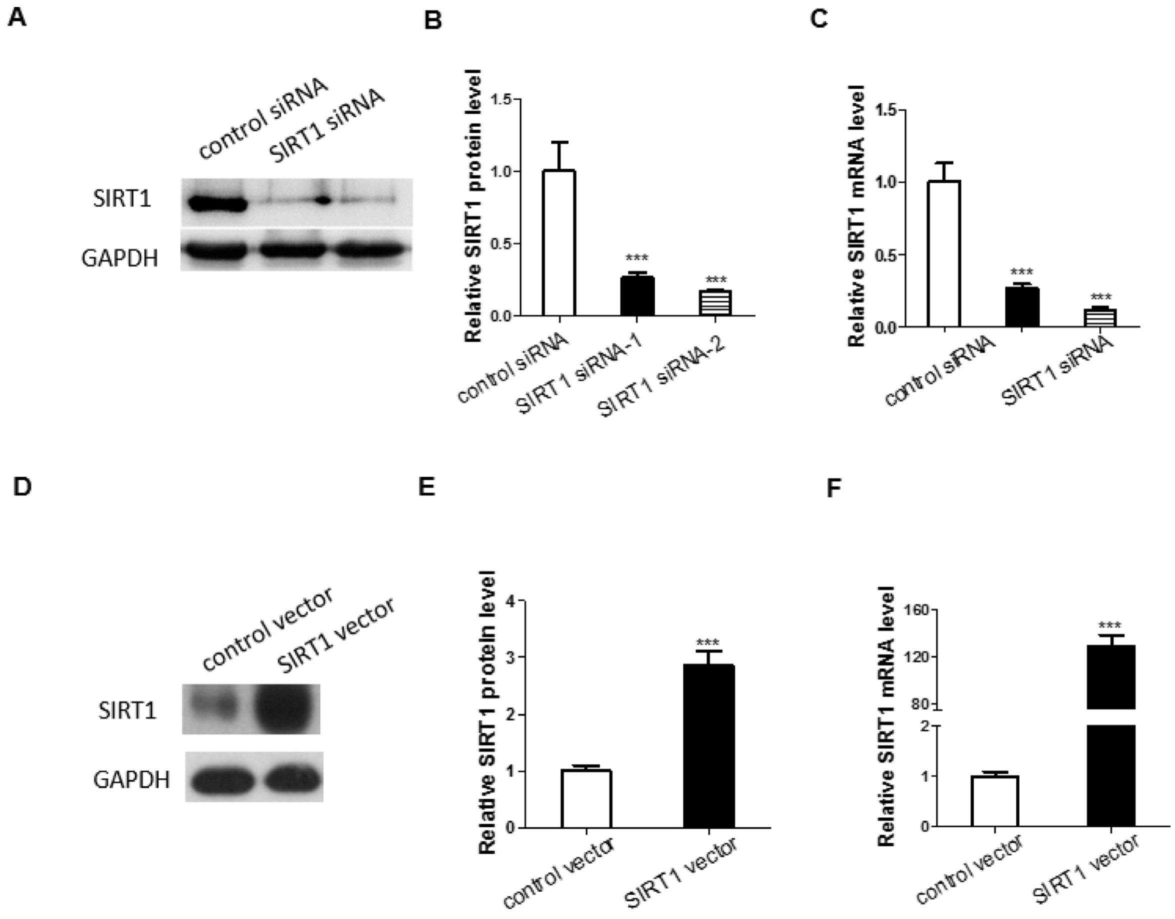

Supplementary Figure 1: Downregulation of SIRT1 by siRNA and upregulation of SIRT1 by an overexpression plasmid in A549 cells. (**A**) Quantitative RT-PCR analysis of SIRT1 mRNA levels in A549 cells transfected with three different SIRT1 siRNAs or the scrambled negative control siRNA. (**B** and **C**) Western blotting analysis of the SIRT1 protein level in A549 cells transfected with SIRT1 siRNA or the scrambled negative control siRNA. B: representative image; C: quantitative analysis. (**D**) Quantitative RT-PCR analysis of the SIRT1 mRNA level in A549 cells transfected with the SIRT1 overexpression plasmid or control plasmid. (**E** and **F**) Western blotting analysis of the SIRT1 protein level in A549 cells transfected with the SIRT1 overexpression plasmid or control plasmid. E: representative image; F: quantitative analysis. The results are presented as the mean ± S.E. of three independent experiments. ($^{***}p < 0.005$).

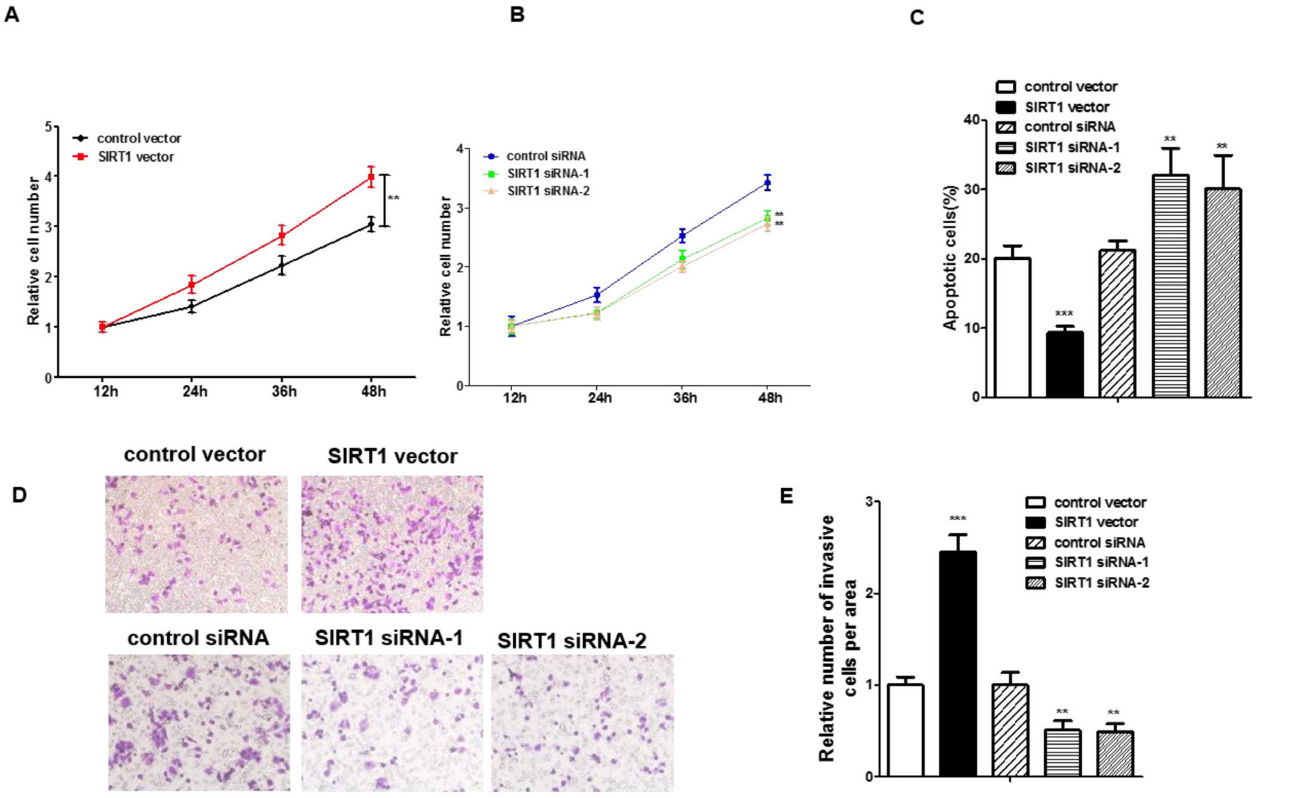

Supplementary Figure 2: Effect of SIRT1 on the proliferation and apoptosis of lung cancer cells. (**A**) The cell proliferation assay was performed after the transfection of A549 cells with the SIRT1 siRNA or scrambled negative control siRNA. (**B**) The cell proliferation assay was performed after the transfection of A549 cells with the SIRT1 overexpression plasmid or control plasmid. (**C**) The apoptosis assay was performed 24 hours after the transfection of A549 cells with equal doses of the SIRT1 siRNA or scrambled negative control siRNA, SIRT1 overexpression plasmid or control plasmid. (**D**) Transwell assay was performed after the transfection of A549 cells with equal doses of the SIRT1 siRNA or scrambled negative control siRNA, SIRT1 overexpression plasmid or control plasmid. (D) representative image; (**E**) quantitative analysis. The results are presented as the mean ± S.E. of independent experience ($^{**}p < 0.01$; $^{***}p < 0.005$).

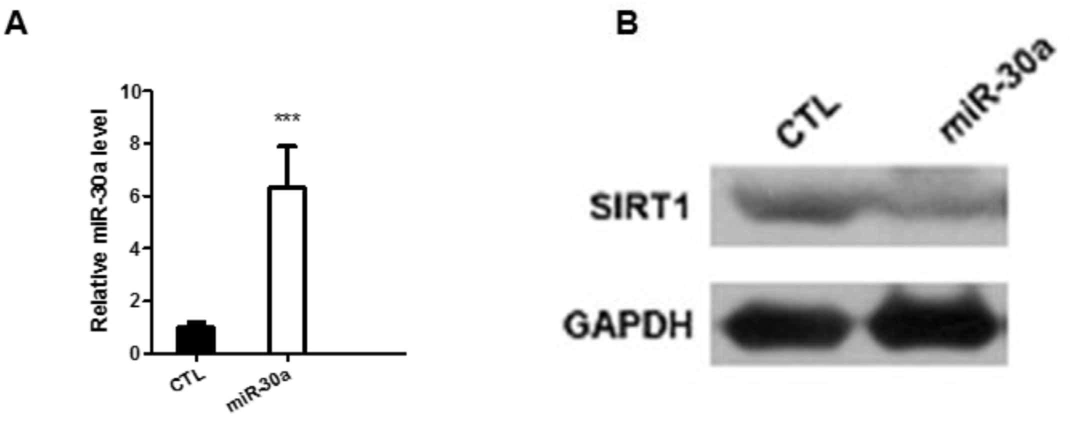

Supplementary Figure 3: Downregulation of SIRT1 by a lentiviral miR-30a expression vector in A549 cells. (**A**) Quantitative RT-PCR analysis of miR-30a, levels in A549 cells infected with a lentiviral miR-30a expression vector. (**B**) Western blotting analysis of HIC1 protein levels in A549 cells infected with a lentiviral miR-30a expression vector. $^{***}P < 0.001$.

Supplementary Table 1: Patients' Characteristics

Patients' characteristics				
**Case No.**	**Tumor subtype**	**Gender**	**Age (years)**	**Pathological Stage**
LC #1	Adenocarcinoma	Male	64	IIA
BC #2	Adenocarcinoma	Female	60	IIA
BC #3	Adenocarcinoma	Male	47	IB
BC #4	Adenocarcinoma	Male	49	IIB
BC #5	Adenocarcinoma	Female	41	IIIA
BC #6	Squamous cell carcinoma	Male	39	IB

Supplementary Table 2: Sequence of 3′UTR and mutant 3′UTR of SIRT1 cloned in reporter vector (miR-30 binding site (MBS) marked in blue)

Sequence of 3′UTR of SIRT1 cloned in reporter vector	ATCGTATTGAATGGCCATTTCCCTACTTATAAGATGTCTCAATCTGAATT TATTTGGCTACACTAAAGAATGCAGTATATTTAGTTTTCCATTTGCATG ATGTTTGTGTGCTATAGATGATATTTTAAATTGAAAAGTTTGTTTTAAA TTATTTTTACAGTGAAGACTGTTTTCAGCTCTTTTTATATTGTACATAGT CTTTTATG
Sequence of mutant 3′UTR of SIRT1 cloned in reporter vector	ATCGTATTGAATGGCCATTTCCCTACTTATAAGATGTCTCAATCTGAATT TATTTGGCTACACTAAAGAATGCAGTATATTTAGTTTTCCATTTGCATG ATGTTTGTGTGCTATAGATGATATTTTAAATTGAAAAGTTTGTTTTAAA TTATTTTTACAGTGAAGACTGTTTTCAGCTCTTTTTATATTGTACATAGT CTTTTATG
